# rAd5 prime/NYVAC-B boost regimen is superior to NYVAC-B prime/rAd5 boost regimen for both response rates and magnitude of CD4 and CD8 T-cell responses

**DOI:** 10.1186/1742-4690-9-S2-O72

**Published:** 2012-09-13

**Authors:** P Bart, Y Huang, N Frahm, S Karuna, M Allen, NK Kochar, S Chappuis, J Gaillard, B Graham, G Pantaleo

**Affiliations:** 1 CHUV, Lausanne, Switzerland; 2 SCHARP, Seattle, WA, USA; 3 HVTN, Seattle, WA, USA; 4 DAIDS, NIAID, NIH , Bethesda, MD, USA; 5 Vaccine Research Center, NIAID, NIH, Bethesda, MD, USA

## Background

HVTN 078 is a randomized, double blind phase 1b clinical trial to evaluate the safety and immunogenicity of heterologous prime/boost vaccine regimens (NYVAC-B/rAd5 vs. rAd5/NYVAC-B) in healthy, HIV-1 uninfected, Ad5 seronegative adult participants.

## Methods

The rAd5 vaccine expressed clade B Gag-Pol and the gp140 of HIV-1 92RW020 (clade A), HxB2/Bal-V3/ΔV1V2 (clade B) and 97ZA012 (clade C). The NYVAC-B vaccine expressed clade B Gag-Pol-Nef and the gp120 of Bx08 (clade B). 80 healthy, HIV-1 uninfected, Ad5 seronegative volunteers, aged 18 to 45 years, were randomized to the placebo arm (n=5) or one of 4 treatment (T) arms: T1 (n=30), 2xNYVAC-B/1xrAd5 (10E10); T2 (n=15), 1xrAd5 (10E8)/2xNYVAC-B; T3 (n=15), 1xrAd5 (10E9)/2xNYVAC-B; T4 (n=15), 1xrAd5 (10E10)/2xNYVAC-B.

Intracellular cytokine staining responses (percent of CD4+ and CD8+ T cells producing IFN-γ and/or IL-2 in response to stimulation with global PTE peptides) were assessed two weeks after the final vaccination.

## Results

For CD4+ T cells, the overall response rates for IFN-γ and/or IL-2 among the vaccinees were 42.9%, 93.3%, 92.3%, and 85.7% for T1-T4, respectively; and the median response magnitudes for positive responders were 0.26%, 0.76%, 0.40%, and 0.76% for T1- T4, respectively. Both response rates (p<0.01) and magnitudes (p<0.03) of CD4+ T-cell responses were significantly lower in T1 compared to the other three treatment groups. For CD8+ T cells, the overall response rates were 65.5%, 73.3%, 76.9% and 85.7% for T1-T4, respectively; and median response magnitudes for positive responders were 0.32%, 0.99%, 1.86%, and 1.65%, respectively. Response rates were not significantly different between groups; however, response magnitudes were significantly lower in T1 compared to the other three arms (p<0.04).

## Conclusion

Priming with rAd5 followed by NYVAC-B boost is superior to priming with NYVAC-B followed by rAd5 boost for both response rates and the magnitude of CD4+ and CD8+ T-cell responses.

